# Computer-aided volumetric assessment of malignant pleural mesothelioma on CT using a random walk-based method

**DOI:** 10.1007/s11548-016-1511-3

**Published:** 2016-12-27

**Authors:** Mitchell Chen, Emma Helm, Niranjan Joshi, Fergus Gleeson, Michael Brady

**Affiliations:** 1 Institute of Biomedical Engineering, University of Oxford, Old Road Campus Research Building, Oxford, OX3 7DQ England; 2 The Churchill Hospital, Oxford University Hospitals NHS Trust, Old Road, Headington, OX3 7LE England

**Keywords:** Malignant pleural mesothelioma, Quantitative tumour measurement, Computed tomography, Image processing, Therapy response assessment

## Abstract

**Objective:**

The aim of this study is to assess the performance of a computer-aided semi-automated algorithm we have adapted for the purpose of segmenting malignant pleural mesothelioma (MPM) on CT.

**Methods:**

Forty-five CT scans were collected from 15 patients (M:F $=$ 10:5, mean age 62.8 years) in a multi-centre clinical drug trial. A computer-aided random walk-based algorithm was applied to segment the tumour; the results were then compared to radiologist-drawn contours and correlated with measurements made using the MPM-adapted Response Evaluation Criteria in Solid Tumour (modified RECIST).

**Results:**

A mean accuracy (Sørensen–Dice index) of 0.825 (95% CI [0.758, 0.892]) was achieved. Compared to a median measurement time of 68.1 min (range [40.2, 102.4]) for manual delineation, the median running time of our algorithm was 23.1 min (range [10.9, 37.0]). A linear correlation (Pearson’s correlation coefficient: 0.6392, $p < 0.05$) was established between the changes in modified RECIST and computed tumour volume.

**Conclusion:**

Volumetric tumour segmentation offers a potential solution to the challenges in quantifying MPM. Computer-assisted methods such as the one presented in this study facilitate this in an accurate and time-efficient manner and provide additional morphological information about the tumour’s evolution over time.

## Introduction

Malignant pleural mesothelioma (MPM) is an aggressive thoracic malignancy that is closely linked to past exposure to asbestos. It is currently responsible for over 47,000 annual deaths worldwide, a number which continues to increase despite legal restrictions limiting the use of asbestos in many countries [1]. MPM is currently the greatest single cause of work-related deaths in the UK [2]. Although the production of asbestos was gradually phased out in the 1980s, the disease’s long latency period, typically ranging from 30 to 40 years, has caused a continuing rising trend of MPM in the country, which is projected to peak in 2020 [3]. Moreover, asbestos is still being harvested and used in the developing world, most notably in China and India, where MPM is rapidly becoming a prominent occupational health concern [4, 5].

**Fig. 1:**
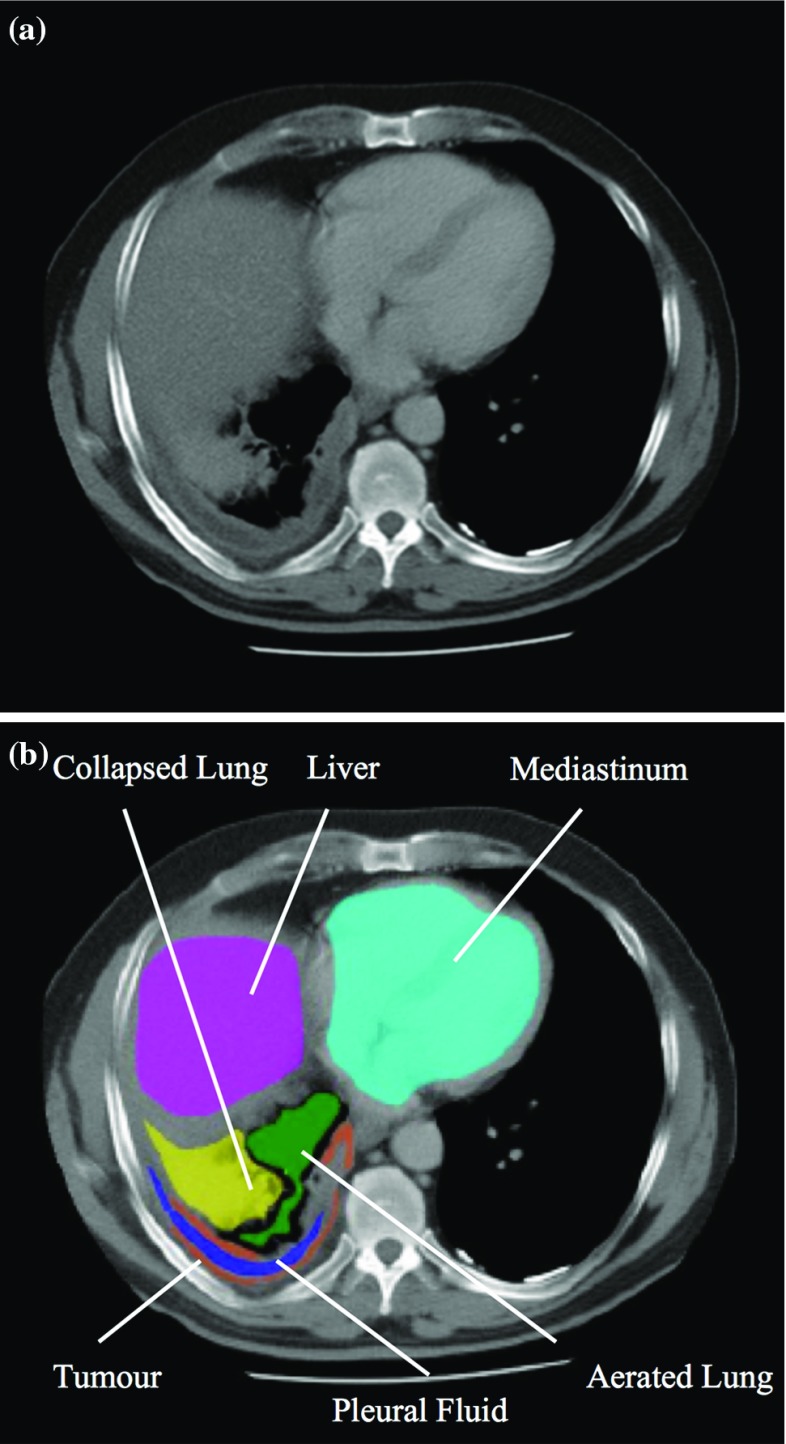
Sample CT image slice with key regional tissues *highlighted*. Tumour is shown in *orange*. **a** Original, **b** segmented CT

**Fig. 2:**
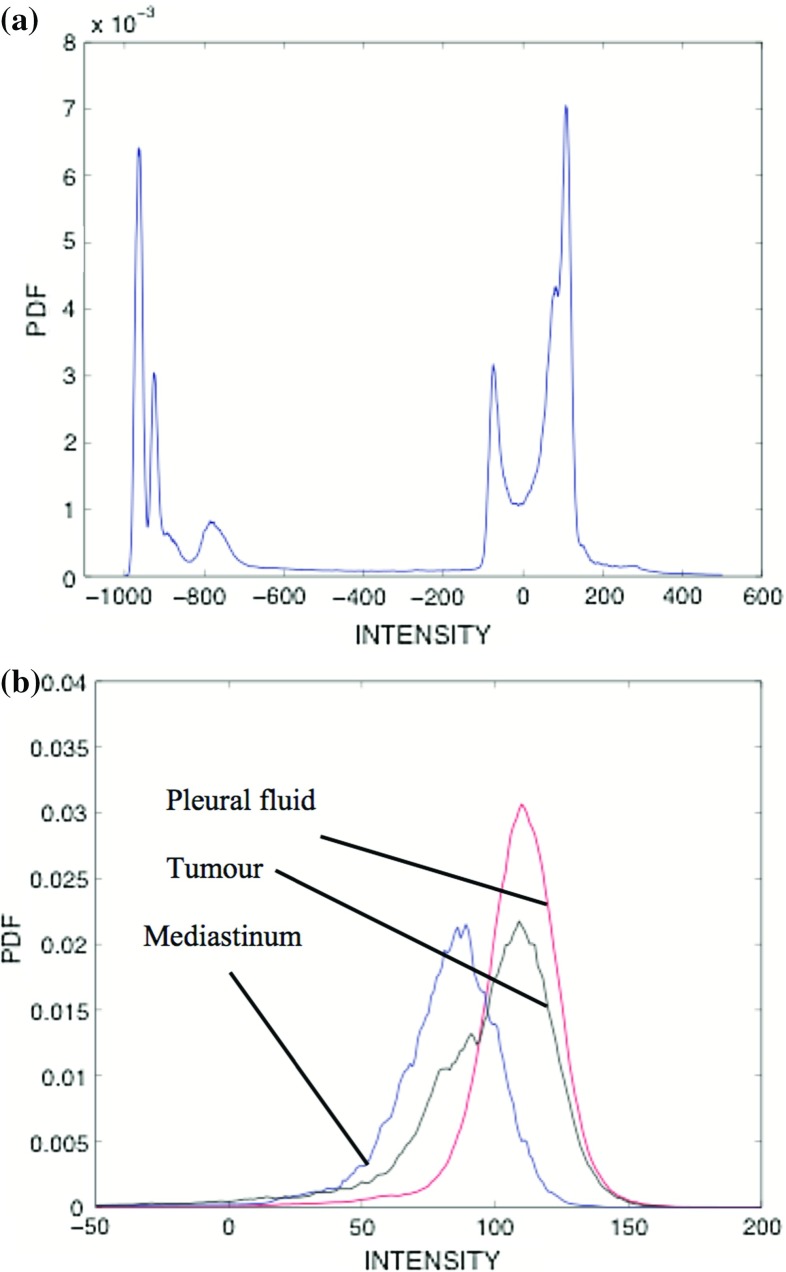
Probabilistic intensity distributions of the overall image scan and individual tissues in the thorax. **a** Overall scan, **b** individual tissues

MPM usually originates in the parietal pleura of the lung and grows as a ‘rind’ around the pleural surface. It has a tendency to encase the affected lung, severely impairing its ventilatory function. The detection of early-stage MPM on CT is difficult because of the complexity of thoracic anatomy and the challenge in distinguishing the tumour from neighbouring tissues, in terms of both pixel intensity and regional texture. A sample CT axial slice, with the relevant thoracic tissues highlighted, is shown in Fig. 1. Intensity distributions of these tissues (in Hounsfield units) are shown in Fig. 2. Their overlapping nature, hence the tumour’s low contrast on CT, is evident.

To quantify the progression of a tumour and its response to treatment in clinical trials and patient follow-ups, current clinical practice recommends the use of MPM-adapted Response Evaluation Criteria in Solid Tumour (modified RECIST) [6]. This quantifies the tumour based on a one-dimensional measurement of its thickness at two locations on three axial levels of the scan. A major drawback of modified RECIST is that it measures the tumour at just six singular points, irrespective of the tumour’s overall shape and growth pattern. As a result, it is known to be prone to intra-observer and inter-observer variations. In one study [7], major and minor disagreements were found in 40% and 10.5% of the cases, respectively. Such discrepancies can largely be attributed to the inconsistent selection of measurable lesions and radiological artefacts such as the partial volume effect, which gives rise to ambiguous tissue boundaries. Additionally, being one dimensional in nature, the system fails to adequately address tumour growth in the axial direction. Overall, considering the low CT contrast between the MPM tumour and surrounding tissues and the tumour’s characteristic ‘rind-pattern’ growth, modified RECIST is limited both in its ease of application, consistency and overall clinical utility.

Noting the limitations of modified RECIST, previous works on MPM [8–16] have supported the use of segmented tumour volumes for evaluating the tumour’s response to treatments.

Ak et al. [8] estimated the tumour volume by counting the number of evenly spaced dots that fall within the tumour boundary, as determined by the clinical observer. Though the results showed good correlation with patient survival and prognostic data, it remains a manual method, which would require tedious and time-consuming radiological supervision.

Armato et al. [9] developed an automated method to compute for modified RECIST. This method works by first segmenting the lungs by thresholding to mark the inner margin of the tumour, followed by taking a thickness measurement perpendicular to the nearby chest wall or mediastinum. The results were presented in terms of ‘clinical acceptance rate’, as determined by trained radiologists, which was found to be as high as 75%. Although this approach offers some guidance for making RECIST measurements, it still makes use of the modified RECIST framework and does not tackle its intrinsic limitations.

In view of the challenging nature of automated MPM segmentation, Frauenfelder et al. [10] applied interpolation to hand-drawn tumour contours found on every 4–5 axial cuts. Inter-observer agreement of the segmented tumour was reported to be significantly higher than that of modified RECIST ($\kappa =0.9$ vs $\kappa =0.33$). However, although interpolation helped reduce the workload, the method did not circumvent the need for manual drawing.

Liu et al. [11] presented some preliminary works on a volume-based MPM tumour evaluation, where the authors studied the baseline and follow-up data collected from 30 patients and found patient survival to be linked to baseline tumour volume with a good degree of certainty.

Chaosaowong et al. [14] introduced a method that computes a contour containing the tumour and pleura. Assuming a convex shape for the pleura, concave irregularities are treated as potential sites of pleural thickening, which would undergo thresholding before the final classification. In addition to being prone to thresholding-related issues, this approach made numerous assumptions about the tumour’s pattern of growth, which would limit its applicability in a wider clinical context.

Sensakovic et al. [15] applied a more advanced MPM segmentation method based on a nonlinear diffusion model and a k-class classifier. For each of the 31 MPM scans examined in the study, 5 axial scans were segmented on a 2-D basis and validated by manual delineations made by five independent clinical observers. Mean Jaccard similarity coefficient (J-index) was found to be 0.517 ($p < 0.05$) between observers and 0.484 ($p< 0.05$) between manual and computed segmentations, which is less than ideal for a wider clinical application. It should also be noted that this method was implemented in 2-D, tested only with diagnostic imaging data, and did not allow the clinician to influence the segmentation itself through user interaction. Labby et al. [16] added an interpolation component to the 2-D method of Sensakovic et al. and extended its scope to MPM follow-up studies. An inverse relationship was reported between the tumour and aerated lung volumes, which is in line with clinical expectation.

It should be noted despite the above efforts, a method that is capable of segmenting all tumour cases accurately is yet to be established.

The segmentation of MPM presents numerous application-specific challenges, one of which is due to the similarity in CT attenuation of an MPM lesion with its neighbouring tissues. This is made worse by the presence of atelectatic lungs and pleural effusion; commonly found in patients with clinically evident MPM. This largely precludes the direct application of simple segmentation methods such as thresholding, region growing, texture filtering, and active contours. The tumour may also grow in finger-like projections along the lung fissures or hilar vessels and can invade the neighbouring structures. This would severely affect the performance of shape and morphology-based methods [13]. Moreover, the long thin shape of the tumour, anatomical complexity of the thoracic region, in particular due to secondary chest conditions such as pleural effusion, intrinsic image noise, and partial volume effect, pose considerable challenges which collectively prevent the application of many established segmentation methods.

In this paper, we present a computer-aided segmentation algorithm that is capable of accurately segmenting the MPM tumour and has the ability to incorporate input from end-users with good robustness.

## Methods and materials

### Study design

The data used in this study were collected from a Phase II clinical trial of Vinflunine, which was tested as a therapeutic agent for MPM across nine centres in the UK, France, and Germany. Specifics on the trial design and outcome are given in [17]. Informed consent was obtained from all individual participants included in the study.

For our study, data from the UK centres were available, collected from 15 patients, with a total of 48 baseline and follow-up CT scans. Patient characteristics are given in Table 1. All patients had histologically and cytologically confirmed cases of mesothelioma and at least one lesion that satisfied the measurability criteria in modified RECIST (defined as pleural tumour thickness of at least 5 mm at three locations on the CT scan, with a sum >20 mm). Each participant received one baseline scan and between one to three follow-up scans. All scans are valid for assessing tumour responses under modified RECIST (i.e. at least four weeks have lapsed between subsequent scans). Patients were treated until either disease progression or unacceptable chemotherapeutic toxicity.

**Table 1:** Patient characteristics

Characteristic	Value
Patient number	15
Mean age (range in years)	62.8 (47.9–79.7)
Male/female [*n* (%)]	10:5 (67:33)
Karnofsky performance status (%)	
80	7 (46.7)
90	7 (46.7)
100	1 (6.7)
Histologic type [*n* (%)]	
Epithelial	12 (80.0)
Mixed	2 (13.3)
Not specified	1 (6.7)
Stage at initial diagnosis (IMIG classification) (%)	
I	1 (6.7)
II	3 (20.1)
III	7 (43.3)
IV	4 (29.9)

**Fig. 3:**
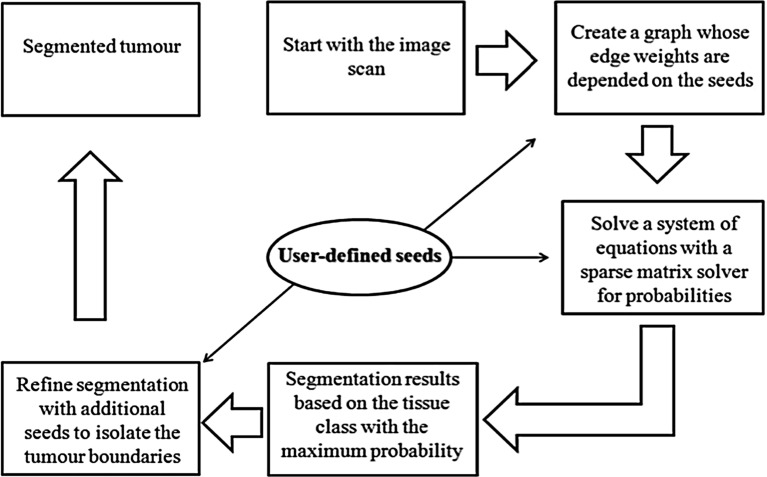
Key steps in the computer-aided method

### Data collection

The CT examinations were performed on a LightSpeed Ultra CT scanner (General Electric Medical Systems). Each CT scan consisted of multiple axial slices of $512 \times 512$ pixels. An assortment of thick (5 mm), thin (2.5 mm), and quasi-isotropic (0.625 mm) scans was available, with a voxel spacing of 0.68 mm. No contrast enhancing agent was used.

### Random walk-based image segmentation

We implemented the semi-automatic random walk-based segmentation method by Grady [18], where details of the mathematical formulation are presented. The method handles weak tissue boundaries well and is able to segment any arbitrary shapes with appropriately placed seeds.

The key steps in our segmentation algorithm are outlined in Fig. 3.

We have extended Grady’s original method to 3-D based on a nine-connected graph model, aiming to improve the algorithm performance by adopting additional image information from adjacent axial slices for classifying the image voxel in question. Performance-wise, it is superior to simple interpolation applied in the vertical axis because uneven tumour growth and anatomical irregularities are dealt with equally in the planar and inter-planar domains and are therefore better accounted for.

Compared to its planar counterpart, a volumetric segmentation would not require exhaustive initialisation on all axial slices; specifying initialisation seeds on just a few axial slices is sufficient to produce accurate full volume segmentations.

### User interaction

User-defined seeds are initialisation points the radiologically trained user places, based on their clinical experience, in regions they known to be of tumour. Using a semi-automated method with user-defined seeds is advantageous because it gives the radiologists more control of the segmentation process by enabling them to influence both the initialisation and post-segmentation revision steps with their clinical knowledge about the patient’s disease.

**Fig. 4:**
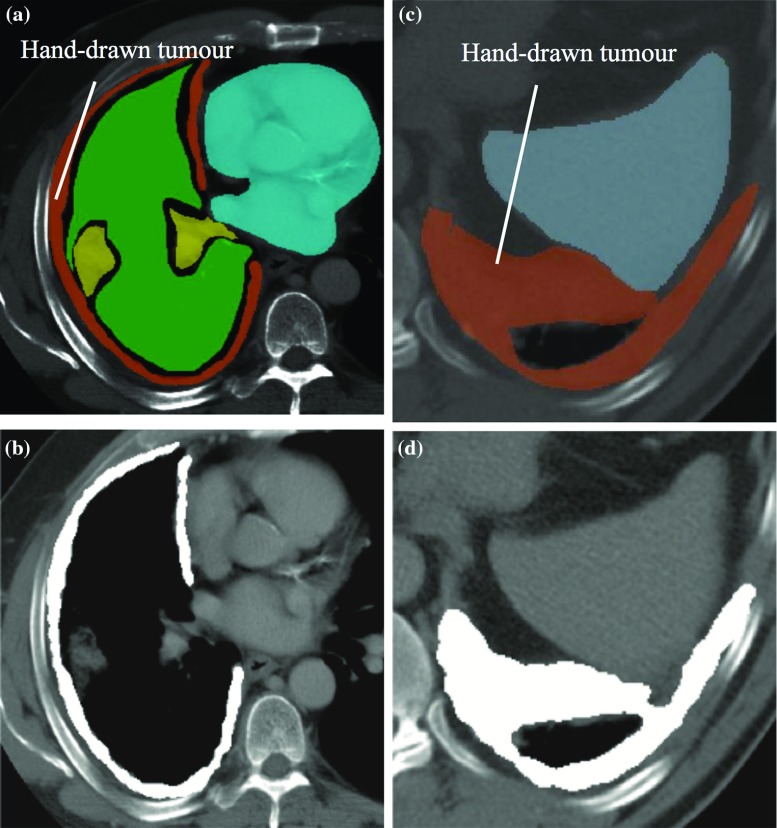
Segmented tumour contours on axial cuts of two arbitrarily selected CT scans, as shown in *white*. Manually delineated tumour contours are shown in *orange*. **a**, **c** Reference truth, **b**, **d** segmented tumour

**Fig. 5:**
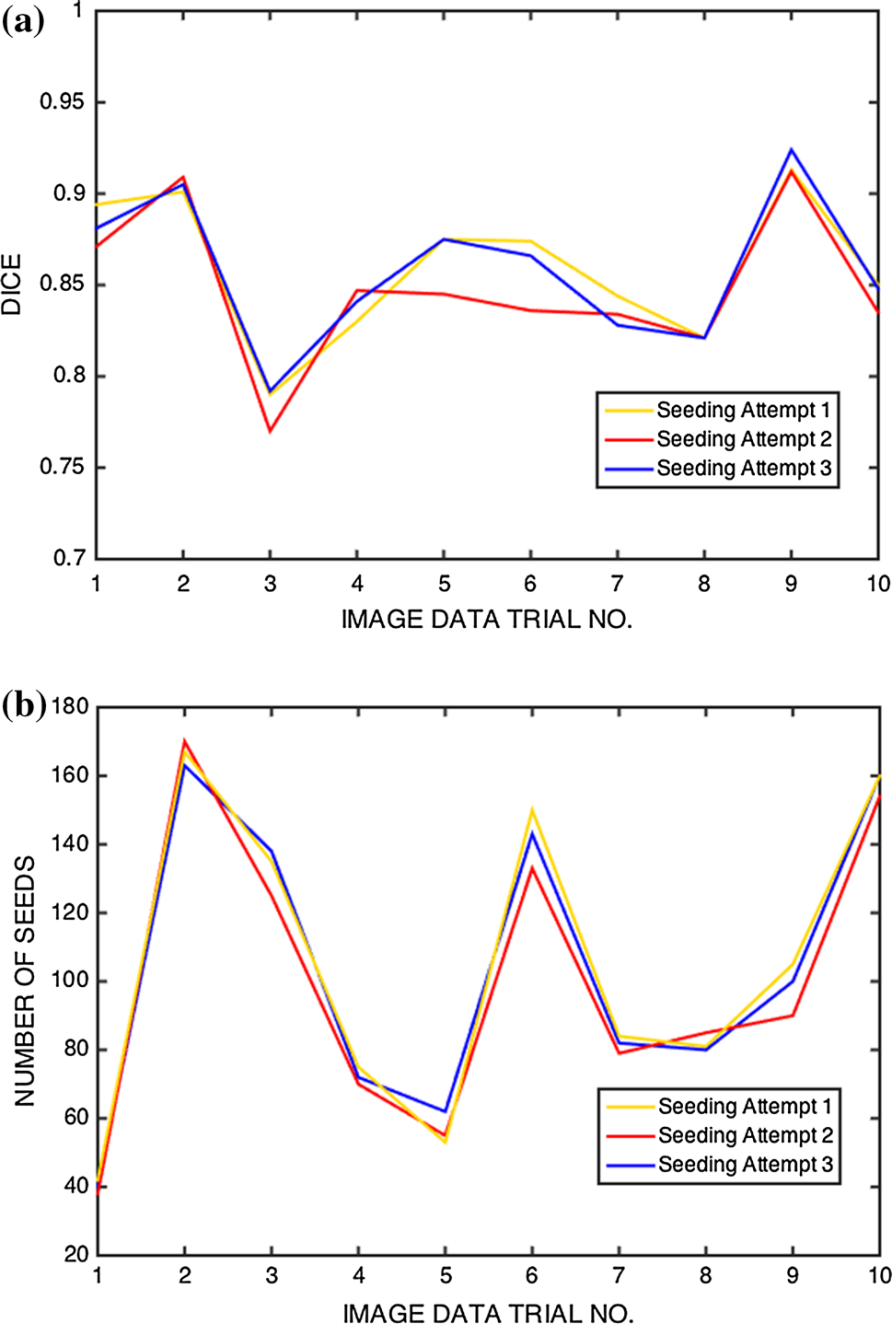
Effect of user input on the method’s performance. **a** Segmentation accuracy in three different seeding attempts, **b** Number of seeds employed in each scenario

**Fig. 6:**
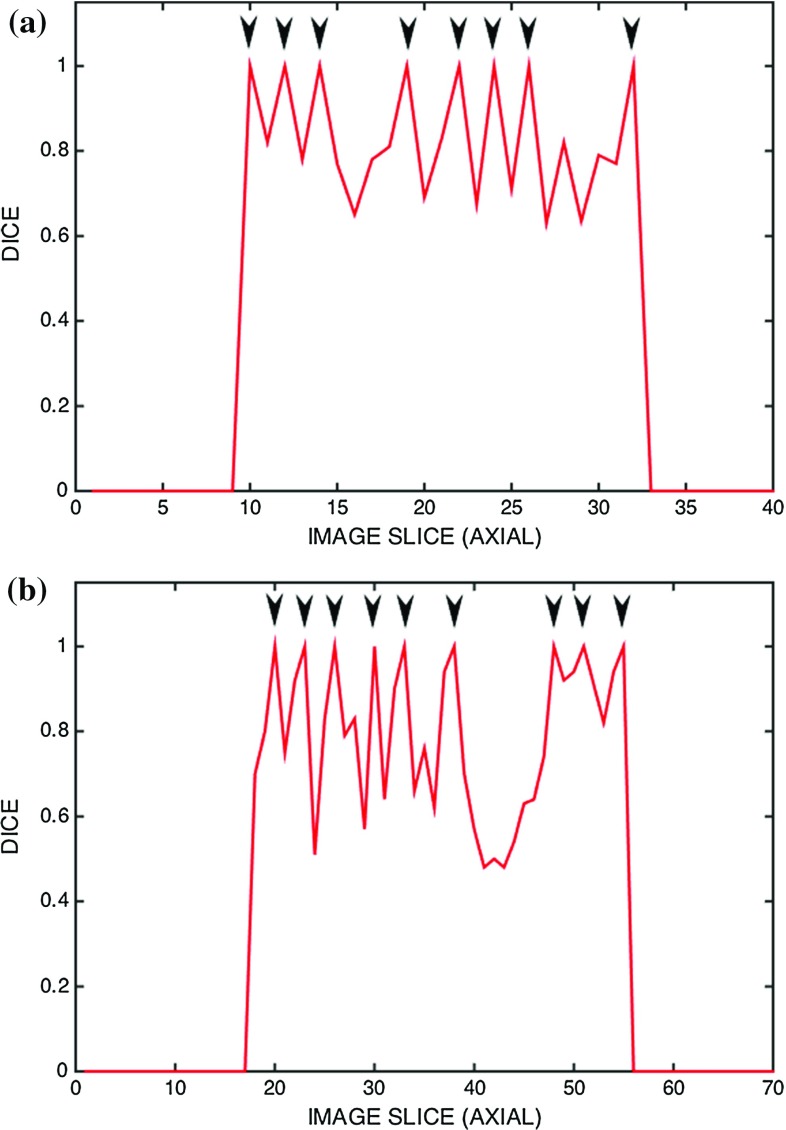
A breakdown of accuracy performance of the two trials presented in Fig. 5. *Arrows point* to the slices where initialisation seeds were placed

However, defining ’seed regions’, contour areas of known tumour instead of points within the tumour, as required by Grady’s random walk-based method, on a large number of image slices is laborious, substantially reducing the clinical practicality of the algorithm. For our study, initialising seeds are placed by a clinical radiology specialist with five years of MPM diagnostic experience, based on expert knowledge about MPM, and added them to between six and ten axial slices for each image scan, that is, one initialised slice for every ten raw image slices. On average, 20–30 seeds were placed per initialisation slice, taking 20–30 s. The initialisation slices were not evenly distributed across the volume scan; they tend to concentrate around regions of difficulty, marked by the presence of weak tumour boundary, fluid and/or collapsed lung. Additionally, to facilitate an effective response evaluation, the same axial locations were selected for initialisation in follow-up scans of the same patient. This is comparable to modified RECIST, where the measurements are taken from the same transverse cuts for different scans of the same patient. Further seeds can be added at a review stage if deemed necessary by the observer, to enhance the segmentation accuracy. This was only required in one out of every three scans. The additional running time for this additional seeding process is minimal, normally a fraction of the overall time spent on the initial segmentation, as will be presented in the Results section of this paper.

All computation times are based on using a workstation with Pentium-D CPU 3.39 GHz with 2GB of RAM.

### Gold standard

To validate our findings, a clinical radiologist with five years of experience in MPM evaluation manually delineated the tumour contours on the whole imaging dataset. Though such delineations vary between observers and over time, for simplicity, we refer to them here as the ’reference truth’. We recognise the fact that the reference truth used might not be ideal, but for the purpose of our study and from an explorative research perspective, we use them here as a surrogate gold standard. To reduce observer bias, a blinded experimental design was implemented, where the delineations were made without knowledge of the clinical outcomes of individual patients and the modified RECIST measurements.

### Data analysis and statistical methods

The accuracy of the segmentation results is evaluated by calculating the Sørensen–Dice index (DICE) according to the following equation, where *X* and *Y* represents the reference truth and the segmentation result, respectively:

1 $$\begin{aligned} \hbox {DICE}=\frac{2\left| {X\cap Y} \right| }{\left| X \right| +\left| Y \right| } \end{aligned}$$

To assess the clinical utility of the computer-assisted method, we computed the correlation of changes in computed tumour volume with those in modified RECIST measurement.

## Results

### Tumour quantification

Applying our computer-aided algorithm, segmented MPM tumour contours are computed in the axial planes. Sample results from two arbitrarily selected scans are shown in Fig. 4. Note the close resemblance of the segmented tumour with the reference truth. The calculated planar DICE measures in these cases were 0.89 and 0.91, respectively.

### Results validation

An assessment of the effect of user input on the method’s performance and its overall robustness is shown in Fig. 5. Note the accuracy of segmentation is largely independent of the number of ’seeds’ used, once beyond a certain level. Note also the good reproducibility and consistency of the results, where changing the ’seeding’ map did not significantly impact on the overall segmentation accuracy.

**Table 2:** Segmented MPM volumes ($\hbox {mm}^{3})$, % change from baseline to the final cycle of treatment and overall accuracy of the segmentation for our complete patient data: mean 0.825 (95% CI [0.758, 0.892])

Patient	Scan				% Change	Overall accuracy (DICE)
	Baseline	2 cycles	4 cycles	6 cycles		
1	229,031	274,025	165,920	182,376	$-$19.1	0.820
2	333,650	289,575	188,461	232,001	$-$30.5	0.843
3	231,266	274,968	285,437		23.4	0.861
4	523,928	338,702			$-$35.4	0.894
5	216,690	165,982			$-$23.4	0.772
6	492,666	180,036	389,923		$-$20.9	0.814
7	348,011	526,396			51.3	0.782
8	315,052	340,443	330,652	291,806	$-$7.4	0.852
9	813,416	870,872			7.1	0.802
10	10,6348	132,937	182,732		71.8	0.791
11	523,828	348,642	397,824	333,911	$-$35.3	0.832
12	939,842	630,513	87,445		$-$7.1	0.840
13	211,220	209,103	152,571		$-$27.8	0.869
14	163,116	208,185	238,774		46.4	0.821
15	476,943	505,001	461,913	634,501	33.0	0.784

**Table 3:** Computation time (s) taken for processing each image scan. The median running time is 1385 s (range [653, 2218])

Patient	Scan				Running time (s)
	Baseline	2 cycles	4 cycles	6 cycles	
1	814	655	684	458	653
2	935	1011	455	815	804
3	2557	780	900		1413
4	1400	1346			1373
5	1474	1147			1311
6	1609	1940	985		1511
7	1466	1314			1390
8	2067	1461	1656	1832	1754
9	1846	1754			1800
10	1063	1233	1543		1280
11	1288	823	579	930	905
12	2538	2491	1624		2218
13	1471	1161	1145		1259
14	1572	1301	1281		1385
15	1623	1721	1476	1716	1634

An analysis of the accuracy measure for individual slices in these cases is shown in Fig. 6. As expected, performance of the algorithm generally deteriorates with distance from the initialisation slices. Full segmentation results and computation times of each individual scan are presented in Tables 2 and 3, respectively.

Good accuracy of the computerised method is clearly demonstrated, with results yielding a mean accuracy of 0.825 (95% CI [0.758, 0.892]).

**Fig. 7:**
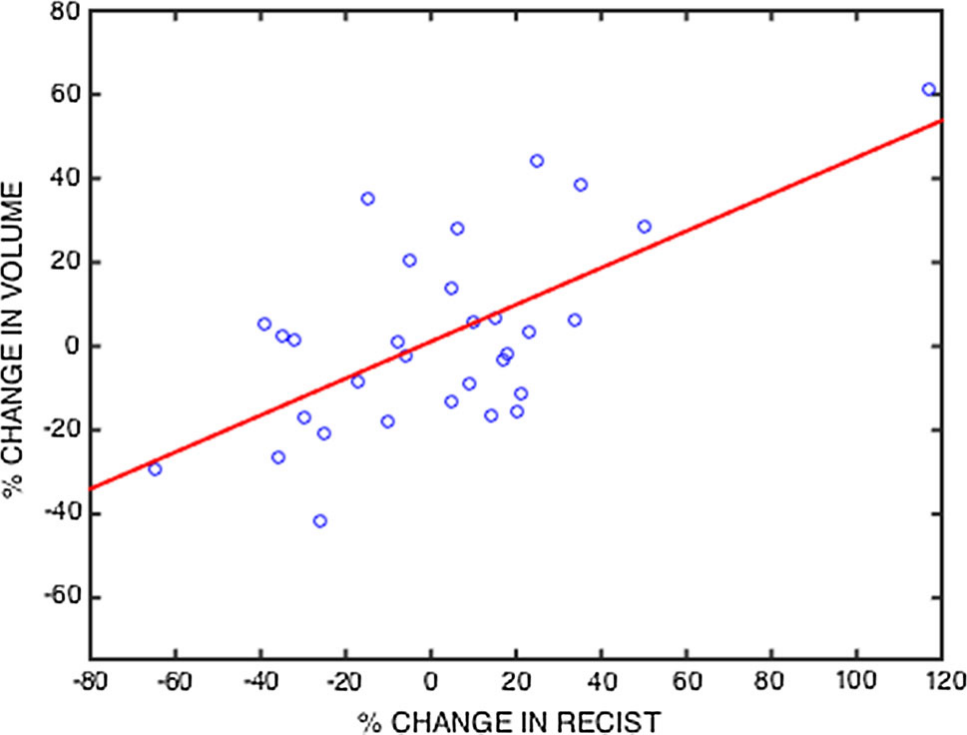
Scatter plots showing the correlation of segmented tumour volumes with their corresponding modified RECIST measures. Pearson’s correlation coefficient 0.6392, *p* value: 0.0001, ${R}^{2} = 0.4086$

**Table 4:** A comparison of results from various published works on computer-assisted MPM segmentation

Study	Study size	Segmentation method	Performance
Ak et al. [8]	57 scans from individual patients	Manual dot counting	Method accuracy not assessed for MPM
Chaisaowong et al. [14]	14 scans from 3 patients	Convex shape with thresholding	Method accuracy not assessed for MPM
Frauenfelder et al. [10]	30 scans from individual patients	Interpolation to hand-drawn tumour contours	Method accuracy not assessed for MPM
Sensakovic et al. [15]	31 scans from 31 patients	Nonlinear diffusion model with k-class classifier	J-index: 0.484 between manual and computed segmentations
Labby et al. [16]	216 scans from 61 patients	Interpolation component added to [15]	Method accuracy not assessed for MPM
Our method	45 scans from 15 patients	Automated random walk	DICE: 0.825 (95% CI [0.758, 0.892])

### Time efficiency of our method

The median time spent on manually delineating the tumour in a typical CT scan was 68.1 min (range [40.2, 102.4]). In comparison, the median running time of the computer-aided procedure was 23.1 min (range [10.9, 37.0]). The median total time spent on manual user interaction was 2.8 min (range [1.8, 4.2]) per image scan. The computer running time could potentially be reduced further by increasing the workstation processing power or by incorporating the use of graphics processor units (GPUs).

Although modified RECIST would take less time to measure from start to finish, it remains a manual process, which requires focused work from a clinician; whereas a computer-aided algorithm such as the one presented in this paper requires little supervision and would enable this clinical time to be invested elsewhere.

### Statistical correlation with modified RECIST

Correlation with the modified RECIST is shown as a scatter plot with linear fit, as shown in Fig. 7.

This shows that tumour response found using the segmented volumes is well correlated with that predicted by the change in modified RECIST and supports the use of volumetric segmentation from a clinical perspective.

## Discussion

Assessing the therapeutic response of MPM is difficult because of the tumour’s poor image contrast and its large variation in shape and growth pattern, which prevent an effective choice of measurement points when employing modified RECIST. These characteristics also happen to make the computer-assisted segmentation of MPM difficult.

It should be noted that apart from Sensakovic et al. [15], most of the existing works on computer-assisted MPM segmentation did not assess the accuracy of their proposed method in a systematic way. Sensakovic et al. [15], whilst presenting a more advanced approach to the problem, produced a less than desirable J-index of 0.484, was implemented only in the planar space, was applied to only diagnostic imaging data, and did not allow the clinician to influence the segmentation itself through user interaction.

Our proposed method, on the other hand, incorporates a three-dimensional framework, flexibly accepts end-user inputs, and produces results that are consistently accurate and with competitive running times.

The results from our method are generally satisfactory, yielding a mean accuracy (DICE) of 0.825 (95% CI [0.758, 0.892]), taking a median running time of 1385 s (95% CI [653, 2218]), greatly reducing the time requirement of an otherwise tedious process. The method also exhibits good robustness because changing the user seed number and configuration did not significant alter the end result. A strong correlation was found between the changes in tumour volume and those in modified RECIST. These results support the use of our algorithm in segmenting MPM for both diagnostic and treatment monitoring purposes. A comparison with results from various published works on computer-assisted MPM segmentation is given in Table 4.

To further assess the applicability of our method in a wider clinical context, it is crucial to develop and validate criteria for categorising tumour changes. Oxnard et al. [19] used geometric models to suggest equivalents of the RECIST classification criteria in the volume space. We note that given the small size and distribution of our data, we are unable to validate these thresholds based on our study because only 1 out of our 15 patients would be classified as ‘progressive disease’ and the rest ‘stable disease’. In addition, although these geometric model-derived thresholds provide good insight into potential volume response criteria, because they are based on standard geometric shapes, they may not lend well to the uneven shapes and irregular growth patterns of MPM. Currently, the issue of volume response criteria remains a contentious area requiring more work.

To further our work, larger patient datasets are needed. It would also be helpful to collect more detailed follow-up clinical data from the patients, such as measures of their quality of life and prognostic data, preferably stratified according to age, gender, and disease stage at diagnosis. These additional clinical data would enable us to better validate our method.

Due to the challenges in assessing the MPM tumour based on CT scans alone, there is a growing interest in employing metabolic imaging in MPM follow-ups [20, 21]. To this end, it might be helpful to examine the possibility of bridging the information obtained from segmented tumour volume to metabolic PET data, such as establishing a correlation between changes in the tumour’s metabolic output and a particular trend of morphological change in its shape.

Currently, modified RECIST remains the standard approach to quantifying MPM in clinical practice, mostly due to its favourable prediction of patient survival and its existing wide acceptance in the radiological community. However, computer-aided volume-based methods, such as the one presented in this paper, are emerging as a new tactic to the clinical problem, for their automated action and better performance consistency.
